# Facile Growing of Ni-MOFs on Ni Foam by Self-Dissociation Strategy for Electrochemical Energy Storage

**DOI:** 10.3390/molecules30030513

**Published:** 2025-01-23

**Authors:** Hongmei Li, Yang Li, Shuxian Song, Yuhan Tian, Bo Feng, Boru Li, Zhiqing Liu, Xu Zhang

**Affiliations:** 1 College of Material Science and Engineering, Shenyang Aerospace University, Shenyang 110136, China; 2 School of Chemical Engineering, Ocean and Life Sciences, Dalian University of Technology, Panjin 124221, China

**Keywords:** metal-organic frameworks, self-dissociation strategy, microbelts, supercapacitor

## Abstract

Metal–organic frameworks (MOFs) with redox metal centers have come into view as potential materials for electrochemical energy storage. However, the poor electrical conductivity largely impedes the potentiality of MOFs to construct high-performance electrodes in supercapacitors. In this work, a self-dissociation strategy has been applied to construct Ni-MOF microbelts on Ni foam (NF), where the NF is used as both a support and a Ni source. The transmission channels between the Ni-MOF and NF are favorable for the charge transport due to the in situ self-assembly of the TPA linkers with the dissociated Ni ions from the Ni foam. The grown Ni-MOF microbelt arrays can offer abundant active sites for redox reactions. The prepared Ni-MOF/NF-s electrode can yield a high capacitance of 1124 F g^−1^ at 1 A g^−1^ and retains 590 F g^−1^ at 10 A g^−1^. This design may offer a controllable protocol for the construction of MOF microbelt arrays on various metal substrates.

## 1. Introduction

With the great progress of electronic devices and electronic vehicles, advanced electrochemical energy storage technologies are urgently being developed [1,2]. Different from batteries with the limited charging–discharging currents due to diffusion-controlled redox reactions, supercapacitors—in which the ions are rapidly transported to the surface of the electrode—exhibit remarkable rate capabilities and a long cycle life [3,4]. Hence, they have received great attention. Apart from carbon-based double electric layer supercapacitors, asymmetric supercapacitors with faradaic pseudocapacitance electrode materials show the desired performance owing to reversible and rapid surface redox reactions [5,6,7].

Metal oxides [8,9], hydroxides [10,11], and sulfides [12,13,14] have been widely studied as electrode materials for supercapacitors. Other than inorganic solids, metal–organic frameworks (MOFs) with a porous structure and tunable functionality also show excellent potential applications for electrochemical energy storage [15,16,17]. The central metal ions of MOFs can perform as active sites for redox reactions and the controllable pore structure can provide open channels for the transmission of the electrolyte [18,19]. For instance, Wang et al. [20] synthesized a Co-based layered MOF, which showed a high specific capacitance, good rate capability, and long-term cycling life in 1M KOH. Guo et al. [21] prepared hierarchical porous Zr-MOFs as electrode materials for supercapacitors, showing a specific capacitance of the prepared HP-UiO-66 of 849 F g^−1^ at a current density of 0.2 A g^−1^. Liu et al. [22] prepared a Mn-based MOF by a simple solution method; due to its 3D structure, it reached an admirable specific capacitance of 832.6 F g^−1^ at 1 A g^−1^. The cuboid Ni-MOF fabricated by He et al. also exhibited a high specific capacitance of 804 Fg^−1^ at 1 Ag^−1^ [23]. However, poor electrical conductivity largely impedes the potentiality of MOFs to construct high-performance electrodes in supercapacitors. To improve the electrical conductivity, hybridizing conductive additives, such as carbon materials [24], conducting polymers [25], and metal foams [26] is an effective strategy. Zhou et al. [24] reported that capacitance performance can be successfully improved by the hybridization of Ni-MOFs with graphene nanosheets. The good electronic conductivity of graphene can enable excellent electron transportation for boosting the performance. Zhang et al. [27] have grown CoNi-MOF nanosheets on carbon fiber paper, which displayed a high specific capacitance of 2033 F g^−1^ at 1 A g^−1^. Ni-MOF nanosheets were grown on Ni foam via a hydrothermal method reported by Wang et al. [26]. The as-prepared Ni-MOF/Ni foam exhibited a high specific capacitance with an outstanding cyclic stability. In addition, a Ni-doped Mn-MOF synthesized by Liu et al. [28] showed a high specific capacitance of 1676.6 F g^−1^ at 1 A g^−1^. However, all these grown processes are conducted by mixing substrates with metal precursors, which may lead to the instability of growth in MOFs and make it difficult to meet the needs of structural control.

Ni foam (NF) is a suitable kind of support skeleton with a large pore structure and high conductivity for the growth of electrode materials [29,30]. It can dissociate Ni ions in an acidic solvent. In this work, we have used NF as a support and Ni as a source by taking advantage of its self-dissociation characteristic to grow Ni-MOF ([Ni_3_(OH)_2_(C_8_H_4_O_2_)_2_(H_2_O)_4_]·2H_2_O) arrays directly. The terephthalic acid and metal Ni can undergo a replacement reaction in this acidic environment with a pH of around 5. In this reaction, the Ni can be oxidated into Ni^2+^ and H^+^ can be reduced to H_2_. The as-prepared Ni-MOF/NF-s exhibits a high specific capacitance of 1124 F g^−1^ at 1 A g^−1^. and a good cyclic stability with a capacitance retention of 79% after 4000 cycles at 10 A g^−1^. Additionally, the assembled hybrid supercapacitor demonstrates the maximum energy density of 38.3 Wh kg^−1^ at the power density of 725 W kg^−1^, indicating the promising practical application in energy storage fields.

## 2. Results and Discussion

**Figure 1:**
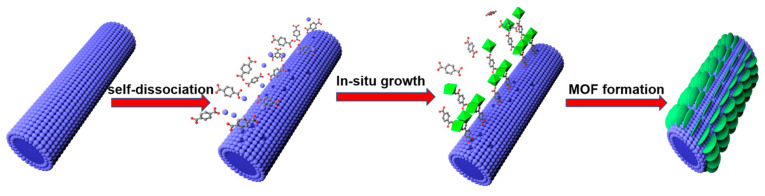
The schematic illustration for the synthesis of Ni-MOF/NF-s.

A schematic illustration for the synthesis of Ni-MOF/NF-s is shown in Figure 1. The NF was immersed in the DMF/H_2_O mixed solvents with dissolved terephthalic acid (TPA) ligands. The pH of the solution measured was around 5. The TPA ligands can release H^+^ in water, which can react with NF to dissociate Ni ions for the formation of Ni-MOF arrays by a one-step hydrothermal reaction.

**Figure 2:**
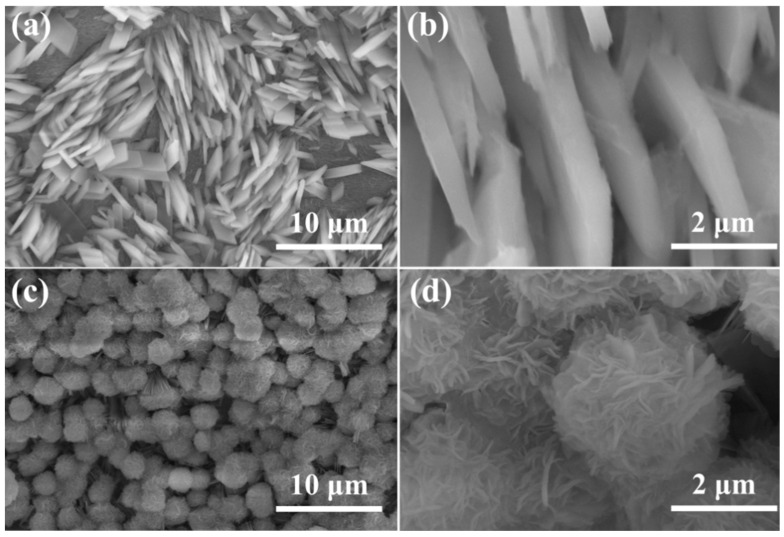
SEM images of (**a**,**b**) Ni-MOF/NF-s and (**c**,**d**) Ni-MOF/NF.

The morphology of Ni-MOF/NF-s and Ni-MOF/NF can be seen in Figure 2. It can be seen that the Ni-MOF was grown on the NF successfully. The formed microbelts are vertically based on the surface and the thickness of the nanosheet is about 170 nm. In addition, the microbelts are assembled into arrays on the NF, which can provide more available surface sites and mass transport channels to improve the electrochemical performance. The morphology of all the Ni-MOF/NF-s is different from the agglomerated nanosheets of Ni-MOF/NF. The difference may be based on whether a large amount of Ni^2+^ ions are present in the liquid phase. If there is a large amount of Ni^2+^ present in the liquid phase, and the MOFs at different locations may be formed simultaneously. But for Ni-MOF-NF-s, the Ni^2+^ ions released from the NF exist on the surface, and the growth of Ni-MOF may be based on the bottom up.

**Figure 3:**
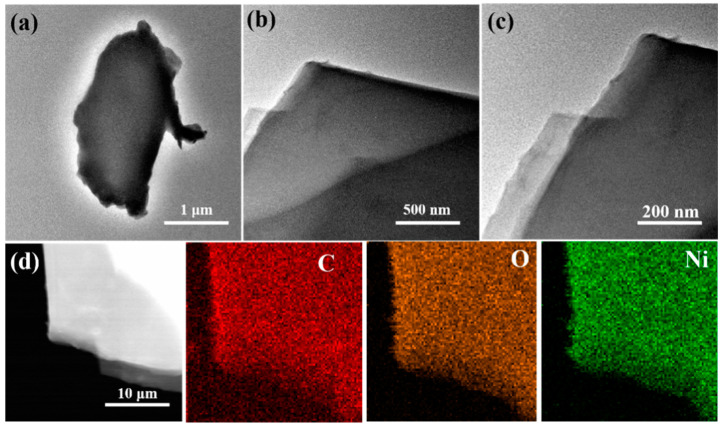
TEM images of (**a**–**c**) Ni-MOF/NF-s and (**d**) the elemental mapping images of Ni, C, and O.

The morphology of Ni-MOF/NF-s is further observed by TEM and scanning TEM, as seen in Figure 3 below, the result of which confirms the 2D structure of the grown Ni-MOF with a smooth surface. Furthermore, the corresponding EDS test from the scanning TEM image shows that the of Ni, O, and C elements are uniformly dispersed through the Ni-MOF microbelts.

**Figure 4:**
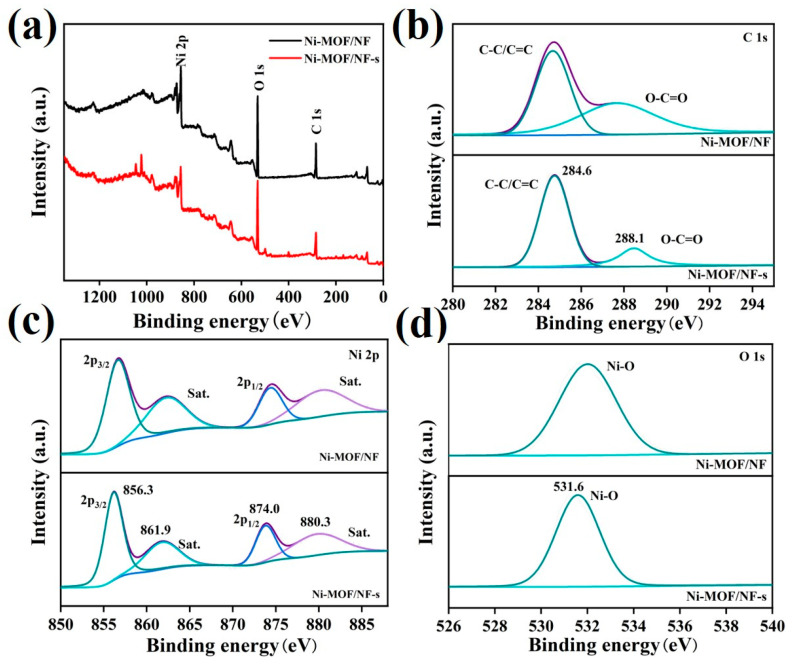
(**a**) XPS survey, (**b**) XPS C 1s, (**c**) XPS Ni 2p, and (**d**) XPS O 1s of Ni-MOF/NF-s and Ni-MOF/NF.

The survey spectra of XPS (Figure 4a) show the coexistence of Ni, O, and C elements in Ni-MOF/NF-s and Ni-MOF/NF. Figure 4b shows similar high-resolution C 1s spectra for Ni-MOF/NF-s and Ni-MOF/NF, where the peaks come from the TPA linkers. The peak at 284.6 eV is related to the carbon of C-C configurations and the peak at 288.1 eV can be related to the carbon of carboxyl groups [31]. Moreover, the high-resolution XPS spectra of Ni 2p of Ni-MOF/NF-s and Ni-MOF/NF are shown in Figure 4c. Ni-MOF/NF-s displays two major peaks at 856.3 (Ni 2p 3/2) and 874.0 eV (Ni 2p 1/2) with their corresponding satellite peaks at 861.9 and 880.3 eV, indicating the divalent state of Ni [32]. Ni-MOF/NF also exhibits deconvoluted Ni 2p 3/2 and Ni 2p 1/2 peaks like those of Ni-MOF/NF-s. Furthermore, in the high-resolution O 1s spectra, both samples show the deconvoluted peaks of Ni-O bonds and O in carboxyl groups. The above results confirm that the composition of Ni-MOF synthesized by the self-dissociation method is consistent with that of the conventional method.

**Figure 5:**
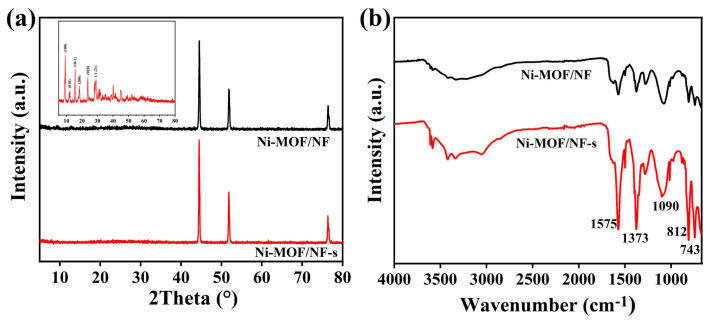
(**a**) XRD and (**b**) FT-IR spectra of Ni-MOF/NF-s and Ni-MOF/NF.

It can be seen from the XRD pattern that all samples have a high intensity peak at about 45°. Because of the high phase strength of nickel foam, the crystal structure of all samples is difficult to find. Therefore, Ni-MOF samples without nickel foam were prepared, and the corresponding XRD characterization is shown in Figure 5a. Significant peaks at 9.2°, 12.1°, 15.6°, 18.5°, 24.0°, and 29.0° (CCDC no. 638866) are similar to reported MOFs synthesized with a metal center Ni^2+^ and the organic ligand TPA [33,34], which can be related to [Ni_3_(OH)_2_(C_8_H_4_O_2_)_2_(H_2_O)_4_]·2H_2_O. An FT-IR spectrograph was used to further analyze the components of Ni-MOF/NF-s and Ni-MOF/NF. The peaks appearing at ca. 743, 812, 1090, and 1373 cm^−1^ are related to the deformation vibration of the aromatic C-H bonds of TPA linkers. Furthermore, the peaks present at ca. 1575 cm^−1^ correspond to the stretching vibration of the -COO^−^ in TPA. In addition, the broad peak at around 3400 cm^−1^ can be assigned to the adsorbed H_2_O molecules [35].

**Figure 6:**
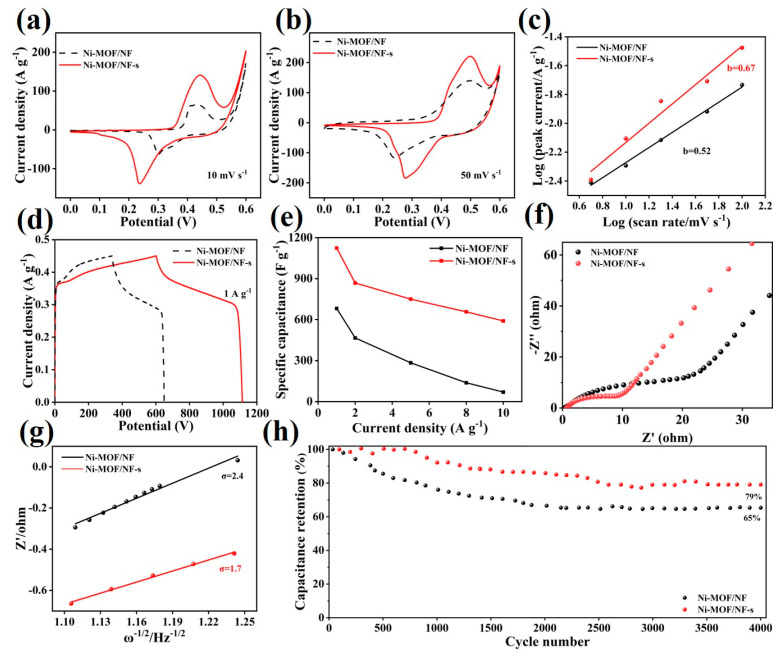
The electrochemical performance of Ni-MOF/NF-s and Ni-MOF/NF: (**a**) CV curves at 5 mV s^−1^, (**b**) CV curves at 50 mV s^−1^, (**c**) b-value determination of the peak currents of CV curves, (**d**) GCD curves at 1 A g^−1^, (**e**) specific capacities at 1–10 A g^−1^, (**f**) Nyquist plots, (**g**) linear relation of Z’ vs. ω^−1/2^, and (**h**) cycling stability at 10 A g^−1^.

The electrochemical properties of the as-prepared Ni-MOF/NF-s are first evaluated in the three-electrode device compared with those of Ni-MOF/NF. The cyclic voltammograms (CVs) curves of various Ni-MOF/NFs at scan rates of 5 mV s^−1^ and 50 mV s^−1^ are displayed in Figure 6a,b. The couple of peaks in the CV curves are attributed to redox reactions of different valence states of Ni, indicating the pseudocapacitive characteristics [36]. Notably, the Ni-MOF/NF-s electrode exhibits larger enclosed curve areas both at 10 and 50 mV s^−1^. This discrepancy indicates a higher reactivity and superior rate capability in the former electrode. The storage energy mechanism is further understood by the equation i = av^b^, where i and ν are the current and scan rate in the CV curves, and a and b are fitting values [37]. A value of b = 0.5 means a diffusion-controlled character, while b = 1 represents surface-induced behavior. In Figure 6c, the b value for Ni-MOF/NF-s is 0.67, higher than the 0.52 of Ni-MOF/NF, and closer to 1, suggesting better surface control behavior. The galvanostatic charge–discharge (GCD) profiles of Ni-MOF/NF-s and Ni-MOF/NF are shown in Figure 6d. The charge–discharge plateaus in the profiles further confirm the pseudocapacitive characteristics of the electrodes. Moreover, the Ni-MOF/NF-s electrode shows a longer discharge time than that of Ni-MOF/NF, implying a better electrochemical performance. In detail, the Ni-MOF/NF-s electrode delivers a higher capacitance of 1124 F g^−1^ at a current density of 1 A g^−1^ than 681 F g^−1^ of Ni-MOF/NF. When the current density is increased to 10 A g^−1^, the Ni-MOF/NF-s electrode still shows a higher capacitance of 590 F g^−1^, maintaining its 52% initial capacitance. The Nyquist plots tested by the electrochemical impedance spectroscopy are shown in Figure 6f, where the semicircle at the high frequency stands for the charge-transfer resistance and the slope at the low frequency reflects ion mobility [38,39]. The Ni-MOF/NF-s electrode has the better ion mobility (higher slope value) and lower charge-transfer resistance (smaller semicircle radius) compared with Ni-MOF/NF. This result is further confirmed by analysis of the ion diffusion kinetic using the equation of Z’ = R_s_ + R_ct_ + σω^−1/2^. Z’ is the real part, R_s_ is the internal resistance, R_ct_ is the charge-transfer resistance, and the ω is the angular frequency. The Ni-MOF/NF-s electrode shows a smaller σ (Warburg diffusion coefficient) value of 1.7 than the 2.4 of Ni-MOF/NF-s and Ni-MOF/NF, as seen in Figure 6g, implying enhanced ion diffusion kinetics. The OH-diffusion coefficient (D) in the electrolyte is calculated by the equation $D=\frac{R^{2}T^{2}}{2A^{2}n^{4}F^{4}C^{2}\sigma^{2}}$, where R (J mol^−1^ K^−1^) relates to the gas constant, T (K) relates to the absolute temperature, A (cm^2^) relates to the electrode area, n is the transfer F (C mol^−1^) which relates to the Faraday constant, and C (mol L^−1^) is the concentration of OH^−^ [40]. The D value of Ni-MOF/NF-s is 4.7 × 10^−17^ cm^2^ s^−1^, which is higher than that of the Ni-MOF/NF material (3.3 × 10^−17^ cm^2^ s^−1^), indicating that the Ni-MOF/NF-s ion diffusion rate is faster, exhibiting excellent ion diffusion/transport dynamics. Since the Ni^2+^ of the Ni-MOFs is coming from the NF, the enhanced structural stability and tight contact interfaces between the Ni-MOF and NF may be favorable for better charge storage and conductivity. In addition, Ni-MOF/NF-s also shows a better cycling stability with a capacitance retention of 79% after 4000 cycles at 10 A g^−1^ than the 65% of Ni-MOF/NF.

**Figure 7:**
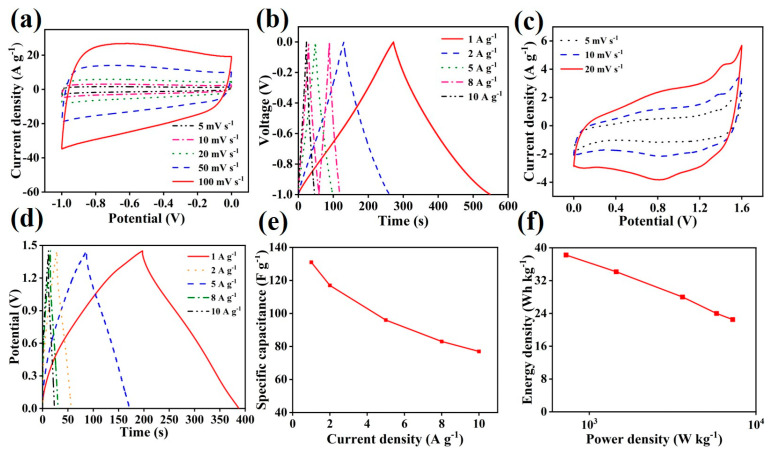
Electrochemical performance diagram of active carbon: (**a**) CV curves and (**b**) GCD curves; and Ni-MOF/NF-s//AC asymmetric supercapacitor: (**c**) CV curves, (**d**) GCD curves, (**e**) specific capacity diagram, and (**f**) Ragone plots.

To estimate the practical application of Ni-MOF/NF-s in the hybrid supercapacitor, Ni-MOF/NF-s is used as a positive electrode and active carbon is used as a negative electrode to assemble the hybrid supercapacitor (Ni-MOF/NF-s//AC). The performance of active carbon can be found in Figure 7a,b. The CV and GCD curves confirm the double layer energy storage characteristic of the active carbon. Figure 7c shows the CV curves of the hybrid supercapacitor at different scan rates (5–20 mV s^−1^), which indicates pseudocapacitance and electric double-layer capacitance in the supercapacitor. The GCD curves at various current densities (1–10 A g^−1^) for the hybrid supercapacitor are shown in Figure 7d, with the nonlinear feature proving the same result as found in CV analysis. The calculated capacitances of the hybrid supercapacitor are shown in Figure 7e. It yields a specific capacitance of 131 F g^−1^ at a current density of 1 A g^−1^ and maintains 77 F g^−1^ at 10 A g^−1^. These results are compared with previously published Ni-MOF based electrodes [17,41,42,43,44,45,46,47], thereby illustrating the excellent performance of our supercapacitor. The Ragone plot shown in Figure 7f shows that Ni-MOF/NF-s//AC has the maximum energy density of 38.3 Wh kg^−1^ at a power density of 725 W kg^−1^, and it can remain at the energy density of 22.5 Wh kg^−1^ when the power density is up to 7250 W kg^−1^. As comparison, Ni-MOF/NF//AC shows a lower specific capacitance of 112 F g^−1^ at a current density of 1 A g^−1^ and maintains a poor performance of 24 F g^−1^ at 10 A g^−1^ (Figure S1).

The cycling test of the Ni-MOF/NF-s//AC can be tested at a current density of 10 A g^−1^, where the 71% of its initial specific capacitance can be remained after 4000 cycles (Figure S2), implying a good cycling performance.

## 3. Materials and Methods

Ni(NO_3_)_2_·6H_2_O (Macklin, Shanghai, China), terephthalic acid (Macklin), ethanol (Fuyu Group, Tianjin, China), and HCl (Xihua Group, Guangzhou, China) were used as received.

NF was firstly cut into pieces with a size of 1 × 3.5 × 0.2 cm. The 0.1 M HCl solution and ethanol were used to wash NF to remove the oxides and impurities. To prepare the Ni-MOF/NF-s, the NF was soaked in 30 mL DMF + 5 mL water solution with 40 mg terephthalic acid (TPA). After sonicating for 30 min, the solution was then transferred into a 50 mL Teflon-lined autoclave and kept at 150 °C for 10 h. After reaction, the product was washed with water and ethanol, as well as dried at 60 °C for 6 h, to obtain Ni-MOF/NF-s.

To prepare the Ni-MOF/NF, the NF was soaked in 30 mL DMF + 5 mL water solution with 50 mg Ni(NO_3_)_2_·6H_2_O and 40 mg terephthalic acid (TPA). After sonicating for 30 min, the solution was then transferred into a 50 mL Teflon-lined autoclave and kept at 150 °C for 10 h. After reaction, the product was washed with water and ethanol to obtain Ni-MOF-s/NF.

## 4. Conclusions

In summary, Ni-MOFs/NF has been easily synthesized through the self-dissociation strategy by using NF as a support and Ni as a source. The in situ self-assembly of the TPA linkers with the dissociated Ni ions from the Ni foam guarantees the unblocked channels of charge transport. The Ni-MOF microbelt arrays can offer numerous active redox sites for improving the electrochemical performance. The as-made Ni-MOFs/NF-s electrode exhibits a high capacitance of 1124 F g^−1^ at 1 A g^−1^ and keeps 590 F g^−1^ at 10 A g^−1^. In addition, the hybrid supercapacitor based on the Ni-MOFs/NF-s shows a high energy density of 38.3 Wh kg^−1^ at a power density of 725 W kg^−1^.

## Data Availability

All data supporting the findings of this study are available within the paper and its Supplementary Materials, which are published online.

## Supplementary Materials

The following supporting information can be downloaded at https://www.mdpi.com/article/10.3390/molecules30030513/s1, Figure S1. Electrochemical performance Ni-MOF/NF//AC asymmetric supercapacitor: (a) CV curves, (b) GCD curves, (c) specific capacity diagram. Figure S2. Cyclic performance diagram of Ni-MOF/NF-s//AC. Table S1. Comparison of the electrochemical properties of Ni-MOF-based asymmetric supercapacitors.
