# Supplementary material for: Facile Growing of Ni-MOFs on Ni Foam by Self-Dissociation Strategy for Electrochemical Energy Storage
Source: Molecules. 2025 Jan 23;30(3):513. doi: 10.3390/molecules30030513 (PMC11819715; doi:10.3390/molecules30030513)
Supplement: Supplementary file 1 [file molecules-30-00513-s001.zip › molecules-3397764-supplementary.pdf]

Facile growing of Ni-MOFs on Ni foam by self-dissociation strategy for electrochemical energy storage

1. Characterization

Field emission scanning electron microscopy (FE-SEM) was carried out on an FEI NOVA NanoSEM 450 system. Transmission electron microscopy (TEM) and energy dispersive spectroscopy (EDS) were taken on an FEI Tecnai G^2 F30 system. The data of X-ray photoelectron spectroscopy (XPS) were collected on a Thermo ESCALAB 250 photoelectron spectrometer. X-ray diffraction (XRD) was used to examine the crystal structure of the obtained samples with 5-80°. Fourier-transform infrared (FT-IR) spectra of the sample were measured using the KBr disk method on a Nicole Avatar 360 FT-IR spectrometer.

2. Electrochemical tests

The electrochemical nature of the samples was tested by a CHI 760E instrument and LAND CT3002A battery test device in a 6 M KOH electrolyte. In the three-electrode system, the working electrode was directly used Ni-MOF/NF-s and Ni-MOF/NF. Meanwhile, a Pt electrode was chosen as the counter electrode and a Hg/HgO electrode was determined as the reference electrode.

The gravimetric capacitance of a single-electrode Cm (F g-1) was calculated according to the galvanostatic charge–discharge profiles. For the three-electrode test,

$$C_m = \frac{I\Delta t}{m\Delta V}$$

where Cm is the specific capacitance (F g-1), I is the discharge current (A), Δt is the discharge time (s), m is the mass of the active material in a single electrode (g), and ΔV is the change in potential during the discharge process (V), respectively.

In the two-electrode system, the device was assembled by using Ni-MOF/NF-s as the positive electrode and active carbon as the negative electrode.

The mass loadings of positive (m+) and negative (m-) materials were determined by the charge balance m−/m+=(C+ΔV+)/(C−ΔV−), where C+ and C- are the capacitances of Ni-MOF/NF-s and active carbon in the three-electrode system.

The C (E, Wh kg-1) and power density (P, W kg-1) were calculated by the total mass of active materials of the two electrodes using the following equations:

$$E = \frac{CV^2}{2 \times 3.6}$$

$$E = \frac{3600E}{t}$$

where C is the specific capacitance of the device, I is the response current (A), t is the discharge time (s), V is the cell voltage, and m is the weight of total active materials on both electrodes (g).

3. Figures

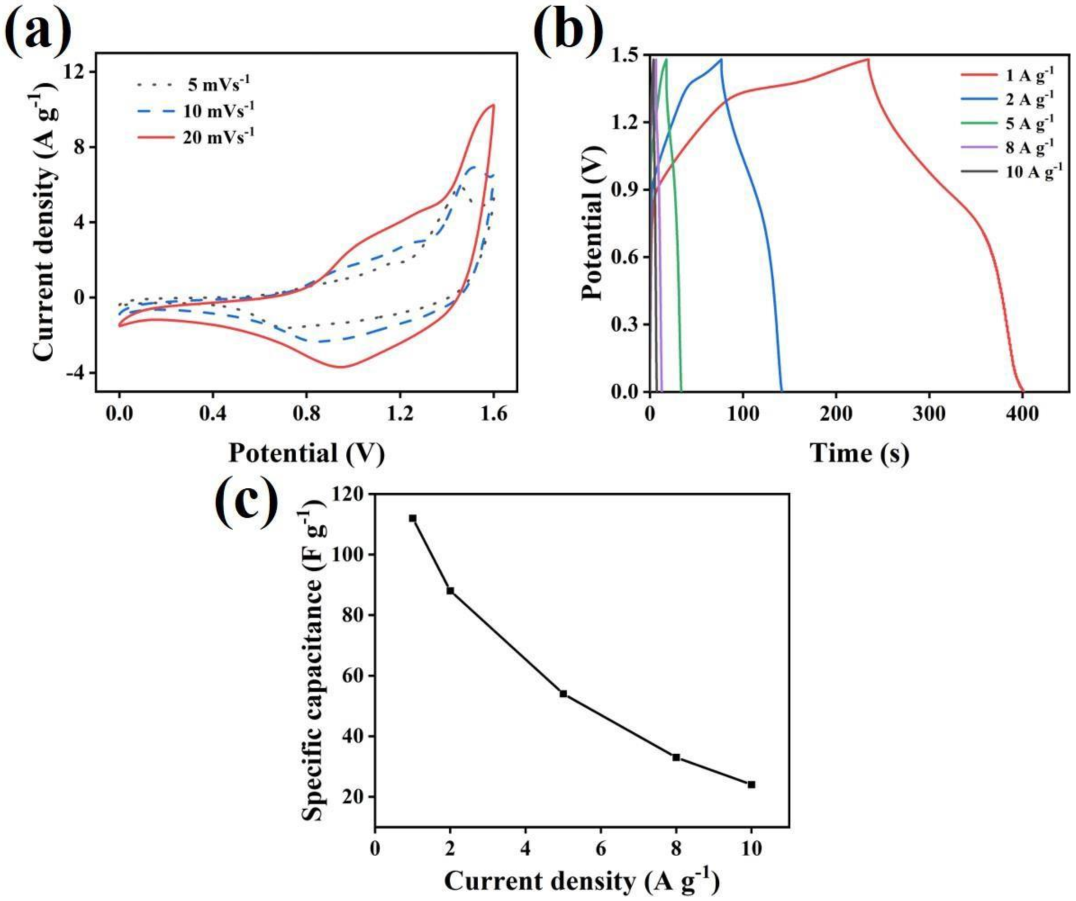

Figure S1. Electrochemical performance Ni-MOF/NF//AC asymmetric supercapacitor: (**a**) CV curves, (**b**) GCD curves, (**c**) specific capacity diagram.

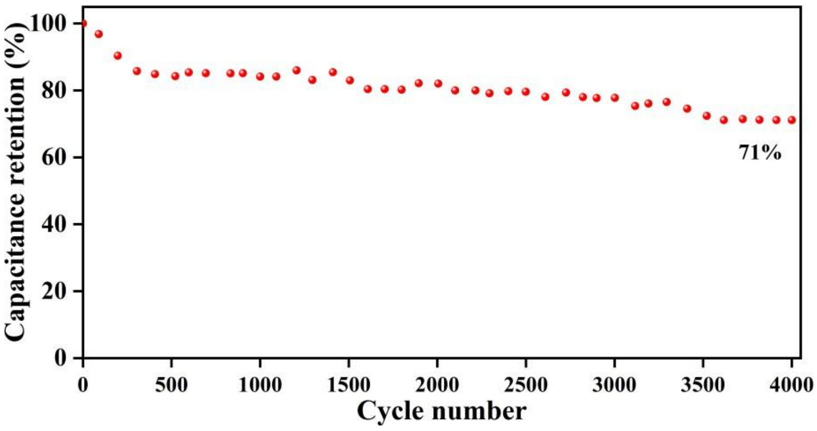

Figure S2. Cyclic performance diagram of Ni-MOF/NF-s//AC.

Table S1. Comparison of the electrochemical properties of Ni-MOF-based asymmetric supercapacitors.

NO.	Sample	Current density (A g^{-1})	Specific capacitance (F g^{-1})	References
1	Ni-MOF/NF-s	1	131	This work
2	3D Ni-MOF	1	66.5	[1]
3	Ni-MOF 1-6	0.5	87	[2]
4	GM-LEG@ Ni-MOF	1	104.5	[3]
5	Ti_3C_2Tx/Ni-MOFs	1	128	[4]
8	NiAl-LDH-0.5/Ni-MOF/S10	1	99.5	[5]
10	Ni-MOF	1	147	[6]
11	Ni-MOF	1	59.4	[7]
12	Co/Ni-MOF-1:15	1	142	[8]

References

1. Wang, J.W.; Ma, Y.X.; Kang, X.Y.; Yang, H.J.; Liu, B.L.; Li, S.S.; Zhang, X.D.; Ran, F. A novel moss-like 3D Ni-MOF for high performance supercapacitor electrode material. *J. Solid. State. Chem.* **2022**, *309*, 122994.
2. Du, P.C.; Dong, Y.N.; Liu, C.; Wei, W.L.; Liu, D.; Liu, P. Fabrication of hierarchical porous nickel based metal-organic framework (Ni-MOF) constructed with nanosheets as novel pseudo-capacitive material for asymmetric supercapacitor. J. Colloid Interface Sci. **2018**, 518, 57-68.
3. Xiao, Y.; Wei, W.; Zhang, M.J.; Jiao, S.; Shi, Y.C.; Ding, S.J. Facile Surface Properties Engineering of High-Quality Graphene: Toward Advanced Ni-MOF Heterostructures for High-Performance Supercapacitor Electrode. *Acs Appl. Energ. Mater*. **2019**, *2*, 2169.
4. Zhang, X.; Yang, S.; Lu, W.; Lei, D.; Tian, Y.; Guo, M.; Mi, P.; Qu, N.; Zhao, Y. MXenes induced formation of Ni-MOF microbelts for high-performance supercapacitors. *J. Colloid Interface Sci.* **2021**, *592*, 95-102.
5. Zheng, W.; Sun, S.; Xu, Y.; Yu, R.; Li, H. Sulfidation of Hierarchical NiAl-LDH/Ni-MOF Composite for High-Performance Supercapacitor. *Chemelectrochem* **2019**, *6*, 3375-3382.
6. Nanda, O.P.; Ravipati, M.; Durai, L.; Badhulika, S. Ni-Metal organic framework nanosheets and Ni_3C/biomass porous carbon composite based long cycle life asymmetric supercapacitor. *Mater. Res. Bull.* **2023**, *168*, 112488.
7. Liang, R.; Du, Y.; Lin, J.; Chen, J.; Xiao, P. Facile-Synthesized Ni-Metal-Organic Framework/Nano Carbon Electrode Material for High-Performance Supercapacitors. *Energ. Fuel,* **2022**, *36*, 7115-7120.
8. Hang, X.X.; Yang, R.; Xue, Y.D.; Zheng, S.S.; Shan, Y.Y.; Du, M.; Zhao, J.W.; Pang, H. The introduction of cobalt element into nickel-organic framework for enhanced supercapacitive performance. *Chinese Chem. Lett.* **2023**, *34*, 107787.
